# Infection with the hepatitis C virus causes viral genotype-specific differences in cholesterol metabolism and hepatic steatosis

**DOI:** 10.1038/s41598-022-09588-w

**Published:** 2022-04-01

**Authors:** David A. Sheridan, Isaac Thom Shawa, E. Louise Thomas, Daniel J. Felmlee, Simon H. Bridge, Dermot Neely, Jeremy F. Cobbold, Elaine Holmes, Margaret F. Bassendine, Simon D. Taylor-Robinson

**Affiliations:** 1 Hepatology Research Group, Faculty of Health, University of Plymouth, Plymouth, PL6 8BU UK; 2 Department of Metabolism, Digestion and Reproduction, Sir Alexander Fleming Building, Imperial College London, South Kensington Campus, London, UK; 3 Research Centre for Optimal Health, School of Life Sciences, University of Westminster, London, UK; 4 Faculty of Health and Life Sciences, Northumbria University, Newcastle-upon-Tyne, NE1 8ST UK; 5 Department of Clinical Biochemistry, Newcastle University Upon Tyne Hospitals NHS Trust, Newcastle-upon-Tyne, UK; 6 Department of Gastroenterology and Hepatology and The NIHR Oxford Biomedical Research Centre, The Oxford University Hospitals NHS Foundation Trust, The John Radcliffe Hospital, Oxford, OX3 9DU Oxfordshire UK; 7 Translational and Clinical Research Institute, The Medical School, Leech Building, Newcastle University, Newcastle upon Tyne, NE2 4HH UK; 8 Department of Surgery and Cancer, Imperial College London, London, W2 1NY UK

**Keywords:** Biomarkers, Diseases, Gastroenterology, Medical research, Pathogenesis

## Abstract

Lipids play essential roles in the hepatitis C virus (HCV) life cycle and patients with chronic HCV infection display disordered lipid metabolism which resolves following successful anti-viral therapy. It has been proposed that HCV genotype 3 (HCV-G3) infection is an independent risk factor for hepatocellular carcinoma and evidence suggests lipogenic proteins are involved in hepatocarcinogenesis. We aimed to characterise variation in host lipid metabolism between participants chronically infected with HCV genotype 1 (HCV-G1) and HCV-G3 to identify likely genotype-specific differences in lipid metabolism. We combined several lipidomic approaches: analysis was performed between participants infected with HCV-G1 and HCV-G3, both in the fasting and non-fasting states, and after sustained virological response (SVR) to treatment. Sera were obtained from 112 fasting patients (25% with cirrhosis). Serum lipids were measured using standard enzymatic methods. Lathosterol and desmosterol were measured by gas-chromatography mass spectrometry (MS). For further metabolic insight on lipid metabolism, ultra-performance liquid chromatography MS was performed on all samples. A subgroup of 13 participants had whole body fat distribution determined using in vivo magnetic resonance imaging and spectroscopy. A second cohort of (non-fasting) sera were obtained from HCV Research UK for comparative analyses: 150 treatment naïve patients and 100 non-viraemic patients post-SVR. HCV-G3 patients had significantly decreased serum apoB, non-HDL cholesterol concentrations, and more hepatic steatosis than those with HCV-G1. HCV-G3 patients also had significantly decreased serum levels of lathosterol, without significant reductions in desmosterol. Lipidomic analysis showed lipid species associated with reverse cholesterol transport pathway in HCV-G3. We demonstrated that compared to HCV-G1, HCV-G3 infection is characterised by low LDL cholesterol levels, with preferential suppression of cholesterol synthesis via lathosterol, associated with increasing hepatic steatosis. The genotype-specific lipid disturbances may shed light on genotypic variations in liver disease progression and promotion of hepatocellular cancer in HCV-G3.

## Introduction

The life cycle of the hepatitis C virus (HCV) is interwoven with lipids at both the hepatocellular stages of virus entry, replication, and assembly (reviewed in^1^) and in the circulation with the formation of complex lipoviral particles (LVP) (reviewed in^2^). Chronic HCV infection (CHC) causes disordered lipid metabolism^3^, and is associated both with lower serum concentrations of low-density lipoprotein (LDL) cholesterol and with hepatic steatosis^4,5^ that resolves following successful anti-viral therapy^6,7^, particularly in those infected by HCV genotype-3 (HCV-G3). Additionally HCV-G3 has been found to be associated with more rapid liver fibrosis progression^8,9^ and an increased risk of developing hepatocellular cancer (HCC)^9,10^, compared to HCV genotype 1 (HCV-G1), independent of patients' age, diabetic status, body mass index, or antiviral treatment. A more recent Korean study of 1448 consecutive CHC patients has proposed HCV-G3 as an independent risk factor for HCC and disease progression^11^. Given the reliance of HCV on host lipid metabolism and clinical challenges posed by HCV-G3 infection, a detailed understanding of lipid perturbation in comparison with HCV-G1 may be relevant for understanding natural history of liver disease progression. Evidence is accumulating that lipogenic proteins are involved in hepatic carcinogenesis^12^ and our previous work has suggested genotype differences in lipoprotein metabolism^13^. In this study, we aimed to further characterise variation in host lipid metabolism between subjects chronically infected with HCV-G1 and HCV-G3.

We have combined several approaches to interrogation of lipid metabolism; initially analysis of the lipidome between subjects infected with HCV-G1and HCV-G3, both in the fasting and non-fasting states, and after sustained virological response (SVR) was performed to identify likely virally-mediated differences in lipid metabolism between the genotypes. Differences in the lipidomes have been correlated with detailed phenotyping of body fat distribution by in vivo magnetic resonance imaging and spectroscopy, including measurements of liver, adipose tissue and intramyocellular fat content in a sub-group of participants.

**Figure 1:**
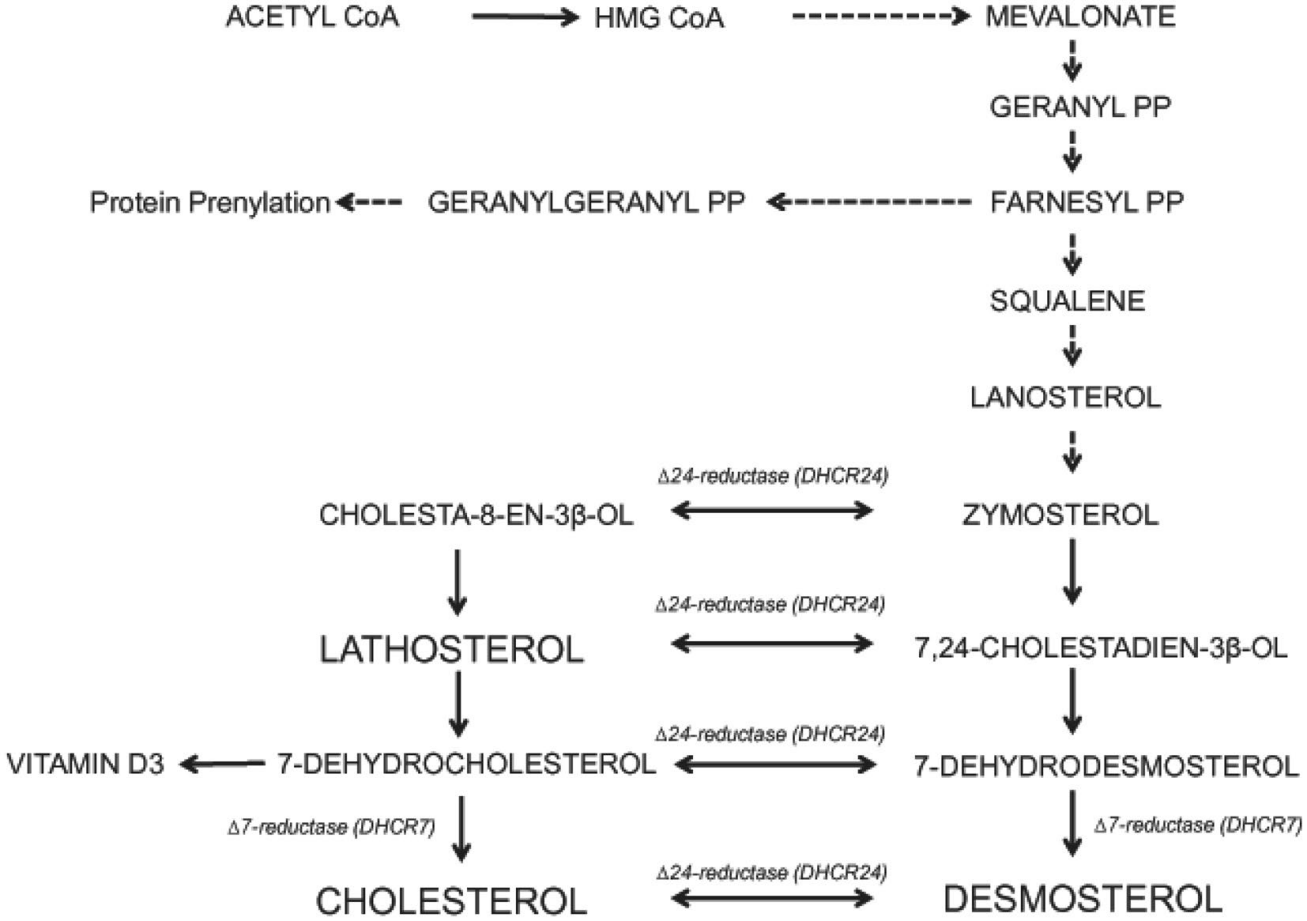
Schematic of the endogenous cholesterol biosynthetic pathway. Cholesterol synthesis involves a complex series of enzymatic reactions from the 2 carbon acetyl CoA to 27 carbon cholesterol. De novo cholesterol biosynthesis takes place in the ER membrane, also the site of HCV replication. The rate limiting step is the activity of 3-hydroxy-3-methylglutaryl (HMG) CoA reductase and the production of mevalonate. The post mevalonate intermediate geranylgeranyl is required for HCV replication. Geranyl that is not used in prenylation is converted to farnesyl and subsequently to squalene, then to lanosterol^18^. From lanosterol, cholesterol biosynthesis can proceed by two routes: via a desmosterol intermediate (Bloch pathway), or via a lathosterol intermediate (Kandutsch-Russel pathway), with flux across the two pathways regulated by ∆24 dehydrocholesterol reductase (DHCR24).

Additionally, for further mechanistic insight into virally-mediated lipid metabolism disturbances, measurement of non-cholesterol sterols in plasma was undertaken; this permitted evaluation of the relative contributions of endogenous cholesterol synthesis and dietary cholesterol absorption to whole body cholesterol homeostasis. Such non-cholesterol sterols are present in small quantities in plasma, distributed and transported with endogenously and exogenously derived cholesterol in all the lipoprotein classes. Lathosterol and desmosterol are late precursors in the endogenous cholesterol biosynthetic (mevalonate) pathway (Fig. 1). Absolute serum lathosterol and desmosterol concentrations and ratios to total serum cholesterol (i.e. lathosterol : total cholesterol ratio and desmosterol : total cholesterol ratio) are an index of endogenous cholesterol biosynthesis^14^ and were also investigated in this study. Sitosterol is a plant sterol, derived exclusively from diet and is therefore an index of intestinal cholesterol absorption^15,16^. Cholestanol is produced endogenously from cholesterol, excreted in bile and then reabsorbed. Serum concentrations of cholestanol reflect cholesterol absorption under physiologic conditions. Cholestanol is increased in cholestatic liver diseases due to decreased biliary secretion^17^. Investigation of these pathways allowed greater insight into the disturbances of lipid metabolism caused by the different HCV genotypes and may allow further insight into the differing propensity for liver cancer development amongst differing viral genotypes.

## Methods

### Patients

Participants with chronic HCV infection were recruited at two centres: Newcastle-upon-Tyne and Imperial College London. All participants gave written, informed consent and the study had ethical approval (Northumberland REC 07/H0902/45 and Fife and Forth Valley REC 07/S0501/21). The research was performed in accordance with the relevant guidelines/regulations set out by the Northumberland and Fife and Forth Valley research ethics committees, and was performed in accordance with the Declaration of Helsinki of 1975.

All participants were age ≥ 18 years, HCV-RNA positive for > 6 months, and not on a lipid modulating agent for 3-months prior to the study. Patients with hepatitis B, hepatitis delta, or HIV co-infection, or alcohol dependency were excluded. All participants attended following a > 8 h overnight fast for sample collection. The fasted cohort consisted of 112 fasting sera (39 G3, 73 G1); 25% had compensated cirrhosis evidenced by Fibroscan > 12.5 kPa (Echosens, Paris, France). Baseline clinical and demographic data are shown in Table 1.

**Table 1:** Clinical and laboratory characteristics of fasting cohort.

Post prandial status	Fasting samples cohort N = 112		
	Fasting > 8 h		*P* value
HCV genotype	1	3	
HCV Viraemic	Yes		
N	73	39	
Age years	48.3 ± 9.9	48.1 ± 10.6	0.918
Male (%)/female	50 (68%)/23 (32%)	30 (77%)/9 (23%)	
BMI (kg/m^2^)	25.4 ± 4.0	25.3 ± 3.0	0.902
Fibroscan LSM KPa Median (Q1-Q3)	7.35 (5.3–16.1)	8.8 (6.5–16.4)	0.143
% Cirrhosis (LSM ≥ 12.5 kPa)	25%	25%	NS
ALT IU/L	96.8 ± 80.5	117.2 ± 68.4	**0.030**
AST IU/L	76.7 ± 63.4	91.2 ± 46.0	**0.022**
Total cholesterol mmol/L	4.62 ± 0.95	3.74 ± 0.91	** < 0.001**
HDL cholesterol mmol/L	1.26 ± 0.36	1.26 ± 0.45	0.953
Non-HDL cholesterol mmol/L	3.36 ± 0.95	2.43 ± 0.82	** < 0.001**
Triglycerides mmol/L	1.31 ± 0.68	1.01 ± 0.72	**0.035**
apoB g/L	0.88 ± 0.26	0.64 ± 0.20	** < 0.001**
apoA1 g/L	1.47 ± 0.29	1.41 ± 0.32	0.328
Fasting glucose mmol/L	5.0 ± 0.69	5.44 ± 1.22	0.095
Fasting insulin μIU/mL	8.07 ± 5.68	7.37 ± 4.11	0.783
HOMA-IR	1.77 ± 1.52	1.88 ± 1.32	0.463
NEFA mM	0.50 ± 0.04	0.54 ± 0.06	0.596

*NEFA* non-esterified fatty acids, *HOMA-IR* Homeostatic Model Assessment for Insulin Resistance.

Significant values are in bold.

In addition, a second cohort of non-fasted serum samples were obtained from the HCV Research UK Clinical Database and Biobank (Glasgow, UK) and comprised 150 treatment naïve chronic HCV patients (75 HCV-G1, 75 HCV-G3), matched to the fasted cohort for age, sex, body-mass index (BMI) and the presence of cirrhosis. A further 100 samples (50 HCV-G1, 50 HCV-G3) were obtained from the HCV Research UK Clinical Database and Biobank from individuals following a sustained virological response (SVR) after successful antiviral treatment (the SVR cohort).

### Liver function tests and serum glucose measurements

Standard serum liver function test and serum glucose measurements were performed on the serum samples from all participants. Aspartate aminotransferase (AST) and alanine aminotransferase (ALT) and serum glucose were measured by standard biochemical methodologies using British National Health Service (NHS) laboratory protocols (https://www.england.nhs.uk/wp-content/uploads/2021/09/B0960-optimising-blood-testing-secondary-care.pdf).

### Fasting lipid profiling

Fasting serum lipids were measured using standard enzymatic methods. Where appropriate, LDL cholesterol was calculated using the Friedewald equation. Apolipoprotein B concentrations were measured by automated rate nephelometric methods (BNII, Dade Behring Ltd, Milton Keynes, Buckinghamshire, UK). Insulin was measured by ELISA (Linco Research Inc, St Charles, Missouri, USA). Lathosterol, desmosterol, cholestanol and sitosterol were measured by gas-chromatography mass spectrometry, (GC–MS), exactly as described previously by Kelley^19^.

### Phenotyping of body fat distribution

A subgroup of 13 consecutively-attending participants from the fasted cohort (6 HCV-G1, 7 HCV-G3) at Imperial College London had additional detailed clinical phenotyping performed by determination of whole body fat distribution using in vivo magnetic resonance spectroscopy (MRS) to quantify intra-hepatocellular lipid (IHCL), intra-myocellular lipids in tibialis (T IMCL) and soleus muscles (S IMCL), and distribution of adipose tissue fat (% visceral and non-visceral fat) using magnetic resonance imaging, as previously described in detail by Thomas and colleagues^20^.

Briefly, 1H MR spectra were acquired from the liver and muscles of the left calf using a surface coil on a 1.5 T Phillips Achieva scanner (Phillips, Best, the Netherlands). Pilot images were obtained to ensure accurate positioning of the (20 × 20 × 20 mm) voxel in the liver (avoiding blood vessels, the gallbladder and fatty tissue) and muscles, ensuring correct placement in the soleus and tibialis muscles. A PRESS sequence (repetition time 1500 ms, echo time 135 ms) was used^20^. Spectra were analysed using jMRUI, with IHCL measured relative to liver water and IMCL measured relative to total muscle creatine^20^. Visceral and non-visceral fat were measured during the same examination. 10-mm thick contiguous axial T1-weighted MR images were obtained throughout the body which were analyzed using SliceOmatic (Tomovision, Montreal, Quebec, Canada).

### Ultra performance liquid chromatography mass spectroscopy (UPLC-MS) lipidomics

All samples were thawed at 4 °C and prepared for UPLC-MS analysis by isopropanol protein precipitation by addition of 150µL of cold isopropanol to each 50 µL serum sample (ratio 3:1), exactly as previously described by Sarafian and colleagues in 2014^21^. Quality control (QC) samples were prepared by pooling equal volumes of all samples and injecting into the mass spectrometry system at regular intervals throughout the analytical runs, in order to define the system suitability, analytical stability, and sample repeatability. Serum lipid UPLC-MS profiling was performed using an ACQUITY UPLC system (Waters Ltd., Elstree, UK), coupled to a Q-ToF Premier mass spectrometer (Waters MS Technologies Ltd, Manchester, UK) using an electrospray (ESI) ion source operated in both positive and negative electrospray ionisation modes (ESI + and ESI-).

Liquid chromatography (LC) conditions have been previously described by Eliasson and colleagues in 2012^22^. Separation was done in a Waters Acquity UPLC HSS CSH column (1.7 μm, 2.1 × 100 mm) maintained at 55 °C. Mobile phases consisted of acetonitrile (ACN)/H2O (60:40) (A) and iso-propyl alcohol (IPA)/ACN (90:10) (B), both containing 10 mM ammonium formate and 0.1% (v/v) formic acid. The flow rate was set at 0.4 mL/min. Injection volume was 5 µL and 15 µL for positive (ESI + ve) and negative (ESI –ve) modes, respectively.

ESI conditions were as follows: capillary voltage for ESI- 2500 V, for ESI + ve 3000 V, cone voltage 25 V for ESI -ve and 30 V for ESI + ve, source temperature 120 °C, desolvation temperature 400 °C, cone gas flow 25L/h, desolvation gas 800L/h. Data were collected in centroid mode. For mass accuracy, leucine enkephalin (555.2692 Da calculated monoisotopic molecular weight) was used as a lock mass. Lock mass scans were collected every 30 s and averaged over 3 scans to perform mass correction. Instrument calibration was performed using sodium formate prior to each ESI mode.

To equilibrate the system, ten conditioning QC samples were performed at the start of acquisition. QC samples were run periodically after 10 sample injections to monitor instrument performance. Data-dependent acquisition (DDA) and MSE analysis of the QC sample was performed to obtain MS/MS information for metabolite annotation. Candidate metabolites were annotated using accurate *m/z* values, fragmentation patterns, retention times, and the METLIN database (https://metlin.scripps.edu/).

### MS data pre-processing

The UPLC-MS raw data were acquired using MassLynx software version 4.1 (Waters, Manchester, UK) and converted in NetCDF files using Databridge; a module within MassLynx software 4.1. The CDF files were pre-processed using XCMS package within the R statistical software version (Rx64 3.2.5), and in-house developed scripts.

### Statistical analysis

Where continuous data were normally distributed, two-sample t-tests were used to compare means between control groups. The Kruskal–Wallis test was used for comparison of non-parametric data. Pearson’s r correlation coefficient was used to determine relationships between continuous variables and Spearman’s rank analysis for correlation between non-parametric variables. *P* < 0.05 was taken to indicate statistical significance. All statistical analyses were carried using Minitab version 16 (Minitab, State College, PA, USA).

### Multivariate statistical analysis

The supervised and unsupervised multivariate models were generated using SIMCA (version 14.1, Umetrics, Umeå, Sweden). Principal component analysis (PCA) and orthogonal projections to latent structures discriminant analysis (OPLS-DA) were performed on all spectral data after pareto-scaling and log transformation for detection of patterns, trends and outliers; and construction of discriminant models were generated for classification and the discovery of potential biomarkers respectively.

### Ethics approval

Ethical approval was obtained from Northumberland Research Ethics Committee (REC 07/H0902/45 and Fife and Forth Valley Research Ethics Committee (REC 07/S0501/21).

### Consent to participate

Prior written, informed consent was obtained from each participant.

## Results

### Clinical phenotype and fasting lipid profiles

Baseline clinical phenotype demonstrated no differences between HCV-G1 and HCV-G3 patients from the fasted cohort in terms of physical demographics of age, sex and BMI or severity of liver fibrosis (Table 1).

Fasting lipid profiles were significantly different in HCV-G3 compared to HCV-G1, manifesting as reductions in total cholesterol, non-HDL cholesterol and apoB (Table 1). Although there was no significant different in fibrosis assessment by liver stiffness, HCV-G3 participants had significantly increased liver enzymes: ALT and AST.

### HCV-G3 decreases cholesterol synthesis via lathosterol rather than desmosterol intermediates

Non-cholesterol sterol intermediates were analysed to understand potential pathways of low cholesterol profiles in HCV-G3. Lathosterol and desmosterol are both pre-cholesterol intermediates, and thus, serum concentrations reflect endogenous cholesterol synthesis. Of note, HCV-G3 patients demonstrated significantly decreased levels of lathosterol, without significant reductions in desmosterol concentrations in serum (Table 2), implying preferential suppression of cholesterol synthesis via lathosterol, with conservation of desmosterol pathway. HCV-G3 patients also had decreased cholestanol concentrations, but no significant difference in the absorption marker sitosterol. This implies decreased biliary cholesterol excretion in HCV-G3, without a compensatory increase in intestinal cholesterol absorption.

**Table 2:** Sterol markers of cholesterol synthesis (lathosterol and desmosterol) and absorption (cholestanol and sitosterol) in fasting sera (cohort 1) (µmol/L).

	HCV genotype 1	HCV genotype 3	*P* value
Lathosterol	3.12 ± 2.12	2.44 ± 1.18	**0.030**
Desmosterol	1.39 ± 0.80	1.37 ± 0.96	0.936
Cholestanol	5.70 ± 2.08	4.78 ± 1.95	**0.022**
Sitosterol	5.28 ± 2.79	5.47 ± 3.87	0.788

Significant values are in bold.

### HCV-G3 subjects have increased intra-hepatocellular lipid content (steatosis)

Detailed body fat distribution phenotyping with whole body MRS was performed in a subgroup of 13 CHC participants from cohort 1 (6 HCV-G1 and 7 HCV-G3). HCV-G3 subjects demonstrated significant increases in IHCL, compared to HCV-G1 infected individuals (5.7 vs. 1.7 µmol/L; *P* = 0.003, Table 3), without other significant changes in IMCL in either soleus or tibialis muscles or adipose tissue compartments (Table 3).

**Table 3:** Subgroup with whole body MRI fat quantification.

	HCV genotype 1	HCV genotype 3	*P* value
N	6	7	
Age (years)	49.8 ± 7.7	54.0 ± 9.2	0.394
Male/female	4M/2F	4M/3F	NS
BMI (kg/m^2^)	25.7 ± 3.3	24.2 ± 3.2	0.420
IHCL	1.7 (0.7–3.3)	5.7 (2.9–7.6)	**0.033**
S IMCL	12.9 (10.7–17)	10.3 (8.69–17.5)	0.609
T IMCL	4.25 (3.3–8.0)	3.36 (1.96–7.11)	0.635
% Visceral adipose tissue	4.25 (1.73–6.25)	2.27 (1.34–4.51)	0.704
% Non visceral abdominal adipose tissue	2.69 (2.16–4.50)	3.47 (2.75–4.27)	0.950

*BMI* body mass index, *IHCL* intrahepatocellular lipid, *S IMCL* intramyocellular lipid, *T IMCL* tibias intramyocellular lipid.

Significant values are in bold.

### Steatosis in HCV G3 does not correlate with markers of VLDL export

**Figure 2:**
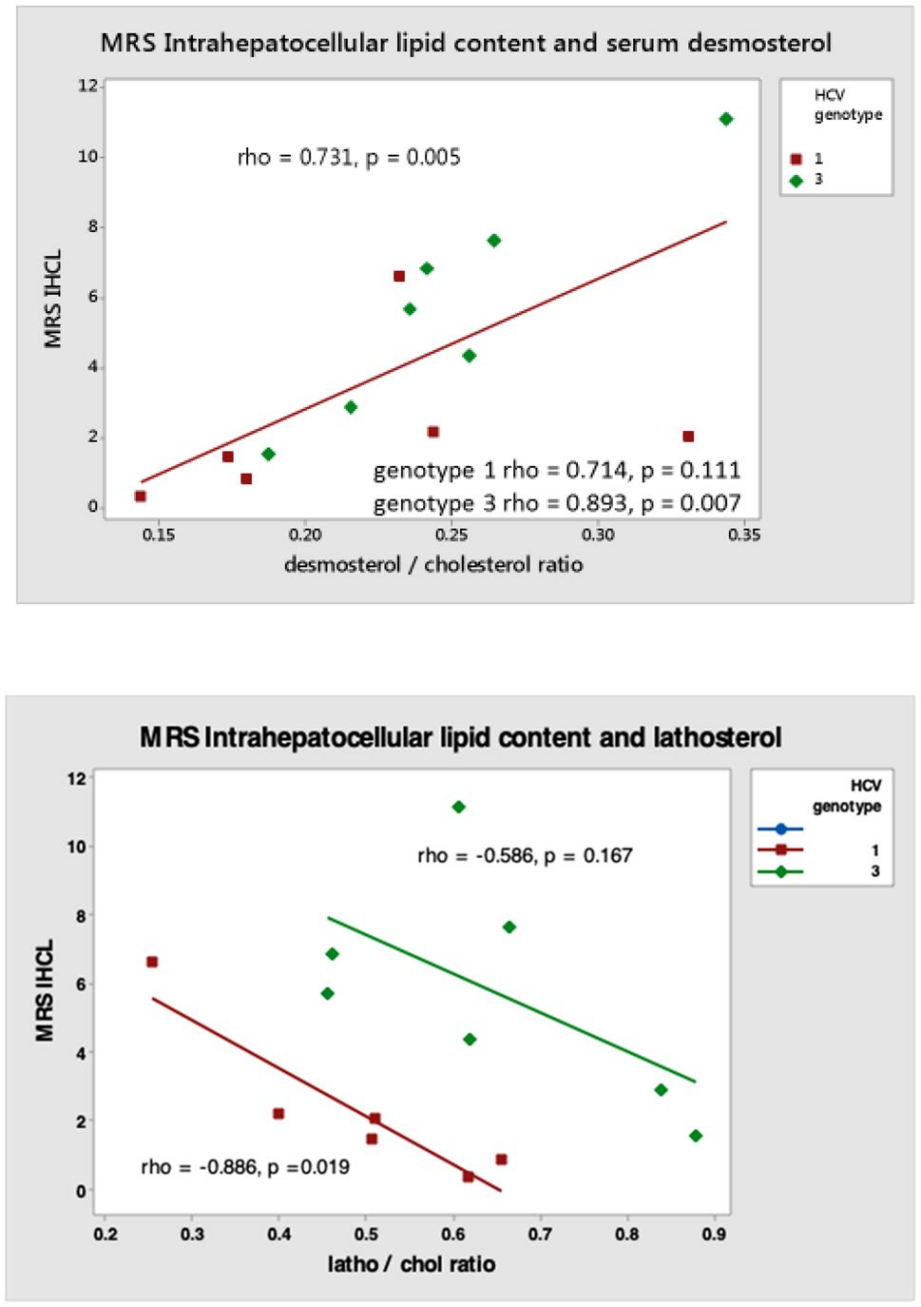
Correlation between intra-hepatocellular lipid content (steatosis) and fasting serum markers of endogenous cholesterol synthesis lathosterol and desmosterol in HCV genotypes 1 (N = 6) and 3 (N = 7).

**Figure 3:**
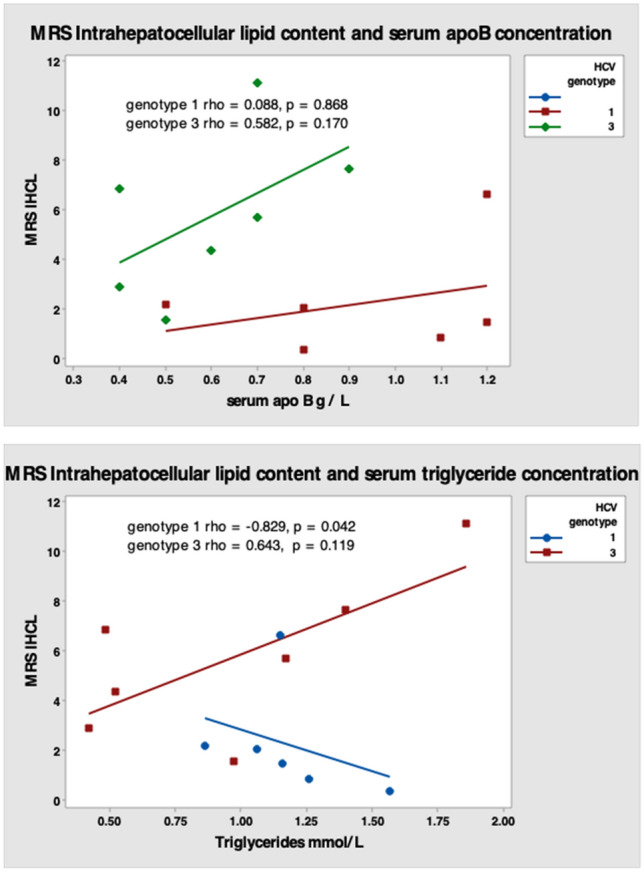
Correlation between intra-hepatocellular lipid content (steatosis) and fasting serum apoB concentration and triglycerides in HCV genotypes 1 (N = 6) and 3 (N = 7).

We explored the relationship between MRS intra-hepatocellular fat content [IHFC] (steatosis) and markers of cholesterol synthesis (lathosterol and desmosterol). IHFC showed a negative correlation with cholesterol synthesis via lathosterol in both HCV-G1 and HCV-G3, which was most marked in HCV-G1. However, we showed a positive correlation with desmosterol in both HCV-G1 and HCV-G3, which was most marked in HCV-G3 (Fig. 2). Serum apoB concentration demonstrated weak positive correlations with IHFC in both HCV genotypes (Fig. 3). There was a negative correlation between steatosis and fasting serum triglyceride (TG) levels in HCV-G1, with a non-significant positive correlation in HCV-G3. Collectively, this implies that steatosis in HCV-G3 is unrelated to decreased very low density lipoprotein (VLDL) particle export, but is more related to viral suppression of cholesterol synthesis via lathosterol, and relative sparing of desmosterol.

### Non-targeted ‘shotgun’ lipidomics identified novel lipid species differentially regulated between HCV-G1 and HCV-G3

**Figure 4:**
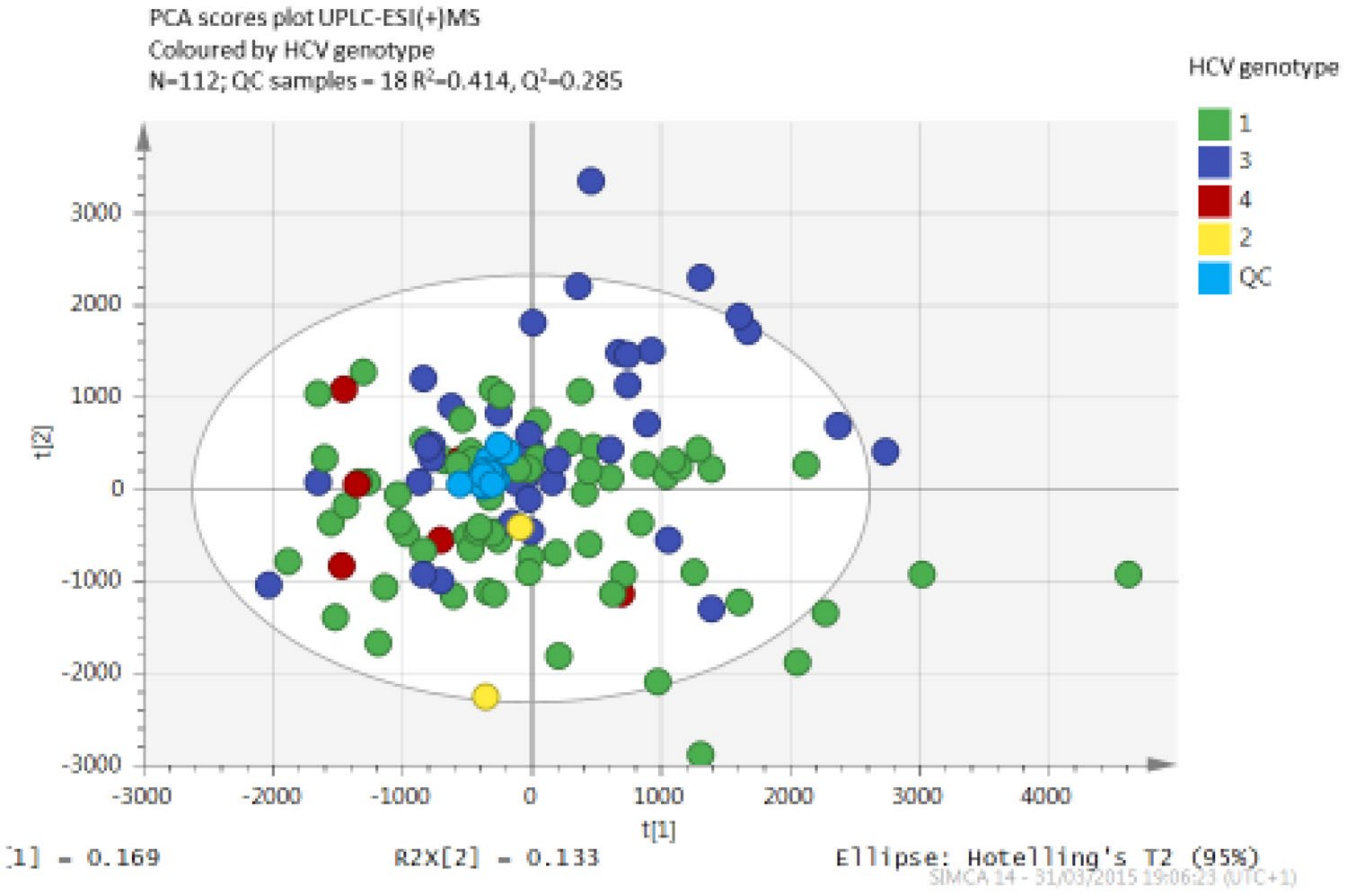
Principal component analysis (PCA) of fasting sera in positive electrospray ionisation mode demonstrating separation between HCV genotypes.

The UPLC-MS spectra from fasting sera of participants in the fasting CHC cohort were explored by PCA to detect clusters and outliers. Pairwise OPLS-DA established the lipids with the strongest contribution to genotypic separation. Figure 4 shows a PCA scores plot indicating clustering of HCV-G1 and HCV-G3, with close clustering of QC samples, indicating good platform stability.

Pairwise analysis using OPLS-DA established the lipid species with the greatest contribution to the genotype separation in positive ion mode (Supplemental Fig. 1).

Using the S-plot from the OPLS-DA model, the influence of individual lipid species (high significance and strong contribution to group separation) in the model were examined. Preliminary assignments were based on mass, fragmentation pattern and retention time of the identified lipid species that were up-regulated in HCV-G3. The main up-regulated lipid in HCV-G3 was cholesteryl linoleate [M + NH4] + 666.621 m/z @15.47 min. In contrast, [M + H] + 784.588 m/z @6.29 min phosphatidylcholine (PC) (36:3) was associated with HCV-G1.

Different lipid species ionise preferentially in only one of the MS polarities; for example, triglyceride preferentially ionised in ESI + whereas free fatty acids in ESI-. Thus, additional novel lipid species were found to be differentially up-regulated in HCV-G1 in the analysis in the negative ion mode (Supplemental Figs. 3 and 4). Assignment of lipid species identified phosphocholines: PhC (36:3 and PhC (38:3)) and (PC(36:5 and PC(38:5)) respectively increased in HCV-G1, whereas cholesterol esters were the discriminant features increased in HCV-G3.

Further UPLC-MS analysis was performed on the non-fasted cohort, consisting of samples from 75 each of HCV-G1 and HCV-G3 patients, matched for age, sex, BMI and presence of cirrhosis to the fasted cohort. The fasting or postprandial status of the samples in the second cohort was not known at the time of sample donation to the HCV Research UK biobank. Supplemental Fig. 5 demonstrates the PCA for the second cohort in both positive and negative ionisation modes. Although the models were less robust than in the fasting samples in cohort 1, the separation of HCV-G1 and HCV-G3, based on the positive ionisation mode retained significance in this independent cohort (Supplemental Fig. 5).

### Discriminating features of the lipidome between HCV genotypes 1 and 3 are not apparent following sustained virological response

To determine whether lipidomic differences resolve or persist after successful eradication of HCV following sustained virological response, further analysis was performed on a third cohort of non-viraemic post-SVR samples (SVR = sustained viral response following HCV antiviral treatment). Supplemental Fig. 6 PCA demonstrates that there is no significant separation by previous HCV genotype exposure following SVR. This supports the notion that the observed genotype-specific alterations in the lipidome in chronic HCV infection are due to the presence of active HCV infection and resolve with viral clearance.

## Discussion

This study performed detailed characterisation of lipid metabolism in individuals chronically infected with HCV and demonstrated that there are distinct HCV genotype-specific changes in lipid metabolism that change following SVR. This study is the most comprehensive description of altered lipid metabolism in subjects chronically infected with HCV-G3 to be reported. We have performed a combination of detailed lipid profiling in fasting samples, including sterol markers of cholesterol synthesis and absorption, and additional quantification of liver, muscle and adipose tissue fat content by in vivo MRS and MRI in a small subgroup. We then performed UPLC-MS lipidomics analysis and made comparison between subjects with HCV-G1 and HCV-G3 in two independent viraemic cohorts, and a post-treatment SVR non-viraemic cohort. The findings have demonstrated that individuals chronically infected with HCV-G3 have significantly decreased serum apoB, and non-HDL cholesterol concentrations, in conjunction with more hepatic steatosis than those with HCV-G1. This finding in itself is not new, but our observations challenge the widely held assumption that the steatosis in HCV-G3 is due to impaired hepatic VLDL export, potentially by inhibition of microsomal triglyceride transfer protein (MTP)^23^. If this were the case, we would have expected negative correlations between liver fat with either serum apoB or TG concentrations in participants with HCV-G3. However, instead we observed a positive correlation between both apoB and TG with liver fat content in HCV-G3, and a negative correlation in individuals infected with HCV-G1. TG accumulation has been reported in HCC, compared to tumour adjacent tissue^24^ and hepatic steatosis is known to be linked to HCC in CHC patients^25^, so the mechanism(s) involved in promoting the differences between HCV-G3 and HCV-G1 could be relevant to hepatocarcinogenesis.

HCV-G3 subjects demonstrated an apparent divergence in decreasing markers of cholesterol synthesis, lathosterol and desmosterol. These two pre-cholesterol intermediates are on separate sides of the late cholesterol biosynthetic pathway. It appears that HCV-G3 preferentially decreases cholesterol synthesis via the lathosterol pathway (Fig. 1). The observation of decreased serum lathosterol levels in HCV-G3, with relatively normal desmosterol levels implies that HCV-G3 selectively inhibits the lathosterol arm of endogenous cholesterol synthesis. Low lathosterol levels have been reported in another study of HCV-G2 and HCV-G3 infection, indicating that HCV-G3 selectively perturbs the late cholesterol synthesis pathway^26^, and in HCV-G3 individuals with cirrhosis, low lathosterol was a predictor of virologic relapse following sofosbuvir and ribavirin treatment^27^. We measured only lathosterol and desmosterol as synthesis markers, which provides information about the relative flux through the two pathways, but did not measure additional upstream pre-cholesterol intermediates. Desmosterol is produced from reduction of 7-dehydrodesmosterol by the enzyme ∆7-sterol reductase (DHCR7) in the Bloch pathway. It has been reported that HCV selectively perturbs the late stages of cholesterol biosynthesis in HCV-G2 and HCV-G3, where lathosterol and 7-dehydrocholesterol concentrations were low, but increased following viral clearance, and the proximal metabolite lanosterol was preserved^28^. The present study adds to the literature by reporting low lathosterol concentrations in a larger number of HCV-G1 and HCV-G3 patients with chronic infection. This may contribute to the high prevalence of vitamin D deficiency among HCV patients^29^. Of additional interest is the strong negative correlation between suppressed cholesterol synthesis via lathosterol and increased hepatic fat content. This implies that as HCV suppresses cholesterol synthesis, pathways of hepatic triglyceride accumulation are being activated without diminishing VLDL export, possibly by an up-regulation of compensatory pathways, such as reverse cholesterol transport and liver X receptors (LXR), which are potently activated by desmosterol.

The data from our untargeted lipidomic analyses of the same cohorts of subjects with HCV-G1 and HCV-G3 in the fasting state have identified additional lipid species differentially regulated between the genotypes, causing clear genotype specific separation of fasting sera in the PCA scores plots. Amongst lipid species accounting for the separation, we observed increased phosphocholines in HCV-G1 and increased cholesteryl esters, including cholesteryl linoleate in HCV-G3. These changes in the lipidome were not apparent in patients who achieved SVR following treatment for previous HCV-G1 or HCV-G3 infection, implying that the lipidomic features are mediated by active HCV viraemia.

Experimental data generated from expression of HCV-G3a core protein in Huh-7 cells have previously reported increasing expression of cholesteryl esters, ceramides and glycosylceramides, but not triglycerides induced by the steatogenic HCV-G3 core protein and suggested that viral steatosis may be distinct from metabolic steatosis^30^. In vivo lipoproteins undergo continuous remodelling during their transit in plasma and we have reported that HCV also undergoes remodelling and transfer on to very-low density lipoproteins after a fatty meal^31^. Increased serum cholesteryl linoleate (CL) levels observed in fasting HCV-G3 participants in our study supports the concept that reverse cholesterol transport is also up-regulated in HCV-G3 infected participants. CL is a cholesteryl ester, which is not synthesised in the liver but produced in the reverse cholesterol transport pathway from peripheral tissues by lecithin-cholesterol acyl transferase (LCAT) activity on HDL. LCAT serves to maintain a cholesterol gradient between peripheral tissues and HDL. LCAT activity enriches HDL in CL as the predominant cholesterol ester. CL is subsequently redistributed amongst all apoB lipoprotein classes by cholesteryl ester transfer protein (CETP) activity, mediating CL transfer from HDL to apoB containing lipoproteins as well, which are subsequently trafficked back to the liver^32^. CETP is increased in active HCV infection^33^. Up-regulated reverse cholesterol transport to the liver may be a compensatory homeostatic response to decreased endogenous cholesterol synthesis in HCV-G3 infection.

Phosphatidylcholine (PC) is a highly abundant phospholipid, and functions as a major constituent of cell membranes. PC is a phospholipid with a typical structure of a choline head group and two fatty acids (FA). The FAs vary in carbon chain lengths and double bond saturation thus a PC molecule may have different fatty acid combinations of varying lengths and saturations attached at the sn-1 and sn-2 positions of the glycerol backbone. However, 16-, 18-, and 20-carbon fatty acids are the most common PCs^32^. In vitro*,* a number of intermediates involved in PC synthesis have been shown to be elevated in HCV infected Huh-7.5 cells^34^. In our in vivo lipidomics analysis, we have identified increased levels of long chain (C36 and C38) unsaturated PCs in fasting HCV-G1 participants. The functional importance of this is not known, but these variations in fatty acids may affect membrane fluidity and utility of PC associated fatty acids as a source of liver triglycerides. In sera, PC is associated with all lipoprotein classes, including HDL and LDL. In HDL metabolism, nascent HDL particles produced by the liver contain lipid poor apoA1, which is then secreted from the liver and gathers excess cholesterol and phospholipids from peripheral tissues by ABCA1- or ABCG1-mediated efflux from peripheral tissues. As HDL particles acquire cholesterol from peripheral tissues they increase in size, hence also acquire additional PC from non-hepatic tissues to accommodate the increasing surface area of the HDL particles. PC associated with either HDL or LDL is subsequently efficiently taken up by hepatocytes. Although we have not shown significant quantitative changes in ApoA1, the PC composition of HDL may be altered by HCV-G1 infection. Studies in mice have indicated that PC is a major and under-recognised source of FA delivery to the liver, which can be a quantitatively important source of hepatic triglyceride. Up to one-third of HDL-PC delivered to the liver can be hydrolysed by PLC and subsequently re-esterified to form hepatic triglycerides. The HDL receptor in the liver is SR-B1, which is responsible for selective uptake of HDL-cholesteryl esters. SR-B1 is responsible for 50% of uptake of PC in isolated hepatocytes^35^. HCV has been also demonstrated to utilise SR-B1 as a hepatocyte entry co-factor^36,37^. Therefore, increased flux of PC through the reverse cholesterol transport/SR-B1 pathway into the liver may favour the HCV lifecycle by utilising entry pathways via SR-B1.

The strengths of this study are that the lipidomics analysis in cohort 1 was performed in fasting samples and demonstrated robust models in PCA of HCV genotype separation in the lipidome, allowing identification of several lipid species differentially regulated by HCV-G1 and HCV-G3, respectively. Although the trend was similar, the models were less robust in the second cohort, which could be explained by the fact that the post-prandial status of the serum samples in cohort 2 and 3 were unknown, and it is likely that donations to HCV Research UK included variable numbers of post-prandial samples. Therefore, the contribution of viraemia to alterations in the lipidome apparent in the fasting state could have been masked somewhat in the presence of varying degrees of postprandial lipaemia.

In summary, we have demonstrated that compared to HCV-G1, HCV-G3 infection is characterised by low LDL cholesterol levels, with preferential suppression of cholesterol synthesis via lathosterol, and preservation of desmosterol levels, associated with increasing hepatic steatosis. Lipidomics analysis revealed lipid species associated with reverse cholesterol transport specifically increased in HCV-G3, which may imply genotype-specific lipid mechanisms involved in liver disease progression and promotion of HCC^38^.

## Supplementary Information

Supplementary Information.

## Data Availability

Available from David Sheridan, Hepatology Research Group, Faculty of Health, University of Plymouth, Plymouth, PL6 8DH, United Kingdom; email: david.sheridan@plymouth.ac.uk.

## Abbreviations

ACN: Acetonitrile
ANOVA: Analysis of variance
ALT: Alanine aminotransferase
AST: Aspartate aminotransferase
BCAA: Branched chain amino acids
BMI: Body-mass index
CCA: Cholangiocarcinoma
CETP: Cholesteryl ester transfer protein
CHC: Chronic hepatitis C
CID: Collision-induced dissociation
CL: Cholesteryl linoleate
CT: Computed tomography
CV‐ANOVA: ANOVA of cross‐validated residuals
DDA: Data-dependent acquisition
ESI: Electrospray ionization
ESI−: Electrospray ionisation negative mode
ESI+: Electro spray ionisation positive mode
HCC: Hepatocellular carcinoma
HCV: Hepatitis C
HCV-G1: Hepatitis C genotype 1
HCV-G3: Hepatitis C genotype 3
HDL: High density lipoprotein
HOMA-IR: Homeostatic model assessment for insulin resistance
IHCL: Intra-hepatocellular lipid
S IMCL: Intra-myocellular lipids in soleus muscle
T IMCL: Intra-myocellular lipids in tibialis muscle
IPA: Iso-propyl alcohol
LC-MS: Liquid chromatography mass spectroscopy
LCAT: Lecithin-cholesterol acyl transferase
LDL: Low density lipoprotein
LXR: Liver X receptors
LVP: Lipoviral particles
MRI: Magnetic resonance imaging
MRS: Magnetic resonance spectroscopy
MTP: Microsomal triglyceride transfer protein
NEFA: Non-esterified fatty acids
NMR: Nuclear magnetic resonance
OPLS-DA: Orthogonal projections to latent structures discriminant analysis
PC: Phosphatidylcholine
PhC: Phosphocholine
QC: Quality control
RP: Reverse phase
SVR: Sustained viral response
TG: Triglyceride
TOF: Time of flight
UPLC: Ultra performance liquid chromatography
VLDL: Very low density lipoprotein
