# Supplementary material for: Infection with the hepatitis C virus causes viral genotype-specific differences in cholesterol metabolism and hepatic steatosis
Source: Sci Rep. 2022 Apr 1;12:5562. doi: 10.1038/s41598-022-09588-w (PMC8975940; doi:10.1038/s41598-022-09588-w)
Supplement: Supplementary file 1 — Supplementary Information. [file 41598_2022_9588_MOESM1_ESM.docx]

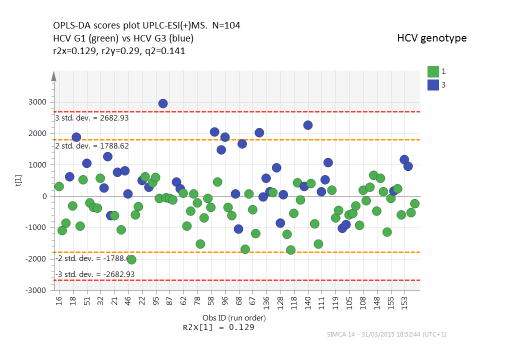


**Supplemental figure 1**. OPLS-DA scores plot to identify those variables with greatest influence on separation of HCV genotypes 1 and 3 in ESI positive mode.


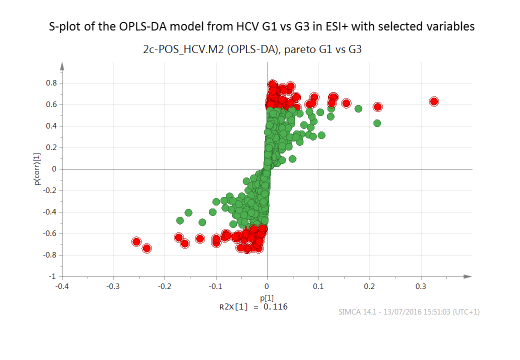


**Supplemental figure 2**: S-plot to determine variables with the greatest contribution to HCV genotypes 1 and 3 separation in fasting samples.


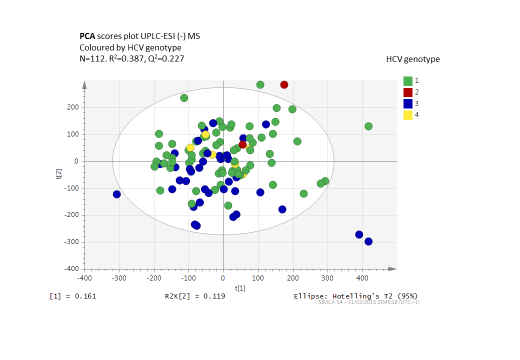


**Supplemental figure 3**. Principal components analysis (PCA) of fasting sera in negative electrospray ionisation mode demonstrating separation between HCV genotypes


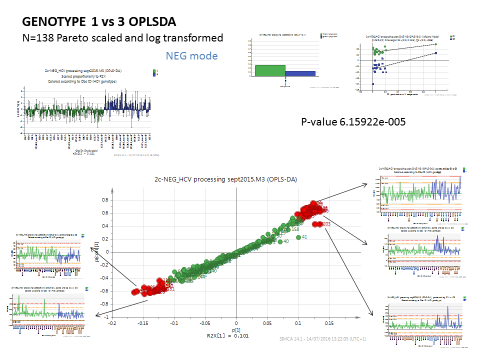


**Supplemental figure 4.** OPLS-DA and S-plot to determine variables with the greatest contribution to HCV genotypes 1 and 3 separation in fasting samples in negative electrospray ionisation mode.


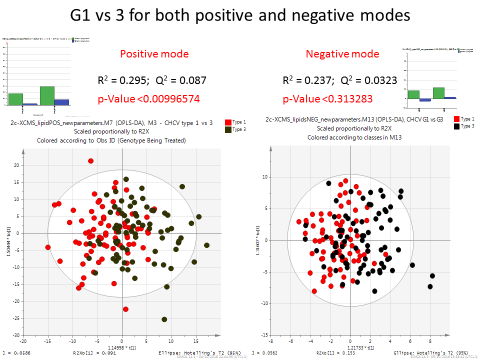


**Supplemental figure 5**. Principal components analysis (PCA) of non-fasting sera, (cohort 2 HCV RNA positive viraemic) in both positive and negative electrospray ionisation mode demonstrating separation between HCV genotypes 1 and 3.


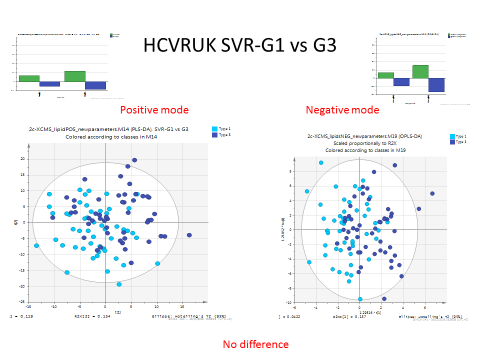


**Supplemental figure 6**. Principal components analysis (PCA) of non-fasting sera, (cohort 3 HCV RNA negative, non-viraemic) following sustained virological response to pegylated interferon-alpha and ribavirin in both positive and negative electrospray ionisation mode demonstrating no significant separation between HCV genotypes 1 and 3.
